# Chafuroside B, an Oolong Tea Polyphenol, Ameliorates UVB-Induced DNA Damage and Generation of Photo-immunosuppression Related Mediators in Human Keratinocytes

**DOI:** 10.1371/journal.pone.0077308

**Published:** 2013-10-08

**Authors:** Tatsuya Hasegawa, Shoichiro Shimada, Hitoshi Ishida, Masaya Nakashima

**Affiliations:** 1 Functional Food Research and Development Center, Shiseido Research Center, Yokohama, Kanagawa, Japan; 2 School of Pharmaceutical Sciences, University of Shizuoka, Shizuoka, Shizuoka, Japan; University of Tennessee, United States of America

## Abstract

Chafuroside B was recently isolated as a new polyphenolic constituent of oolong tea leaves. However, the effects of chafuroside B on skin function have not been examined. In this study, we investigated the protective effects of chafuroside B against UVB-induced DNA damage, apoptosis and generation of photo-immunosuppression related mediators in cultured normal human epidermal keratinocytes (NHEK). Chafuroside B at 1 µM attenuated both UVB-induced apoptosis, evaluated in terms of caspase-3/7 activity, and UVB-induced DNA damage, evaluated in terms of formation of cyclobutane pyrimidine dimers (CPD), in NHEK exposed to UVB (20 mJ/cm^2^). In addition, chafuroside B at 0.3 or 1 µM suppressed the UVB-induced production of interleukin (IL)-10, tumor necrosis factor (TNF)-α, and prostaglandin E2 (PGE_2_), as determined by ELISA, and conversely enhanced IL-12 mRNA expression and production, as measured by RT-PCR and ELISA. Further, chafuroside B at 1 µM also suppressed UVB-induced expression of receptor activator of nuclear factor κB ligand (RANKL) mRNA. These results indicate that chafuroside B promotes repair of UVB-induced DNA damage and ameliorates the generation of IL-10, TNF-α, PGE_2_, and RANKL, all of which are UVB-induced immunosuppression related mediators. These effects of chafuroside B may be mediated at least in part through induction of IL-12 synthesis in human keratinocytes. Because chafuroside B might have practical value as a photoprotective agent, a further study of the in vivo effects of chafuroside B seems warranted.

## Introduction

The skin is the largest organ of the human body and protects the organism against external physical, chemical, and biological insults, including ultraviolet (UV) radiation and microorganisms. Although many environmental and genetic factors contribute to the development of various skin diseases, one of the most important factors is chronic exposure of the skin to solar UV radiation. Excessive exposure of the skin to UV radiation has many biological consequences, including sunburn, hyperpigmentation, solar keratosis, solar elastosis, skin cancer and immunosuppression [1]. UVB (290–320 nm) radiation induces apoptotic cell death of keratinocytes, which are evident within the epidermis as sunburn cells. The formation of sunburn cells in UVB-exposed skin is a measure of the severity of DNA damage. Absorption of UV produces two main types of DNA damage, i.e., formation of cyclobutane pyrimidine dimers (CPD) and pyrimidone photoproducts. However, the repair of DNA damage in UVB-exposed skin cells can prevent the accumulation of damaged cells [2]. UV-induced DNA damage is also an important molecular trigger for UV-induced immunosuppression, as well as various forms of skin cancer [3]. Sensitization to contact allergens was impaired in UV-irradiated skin, and repair of CPD restored the *in vivo* antigen-presenting activity [4], [5]. Although the skin immune system, which is subdivided into the innate and the adaptive immune system, provides a powerful defense against microbial invaders and also plays a role in detecting and eliminating tumor cells [6], some of the adverse effects of UV radiation on human health, including exacerbation of infectious diseases, such as herpes, and initiation of skin cancer, are mediated at least in part by this ability of UV radiation to induce immunosuppression [7]. Indeed, even low doses of sunlight exposure during normal daily activities can suppress immunity in humans [8]. UV-induced immunosuppression is modulated by various cytokines, including tumor necrosis factor (TNF)-α, interleukin (IL)-10 and IL-12 [9]. After UV exposure of skin, TNF-α and IL-10 are released from UV-stimulated keratinocytes [9]. TNF-α is a proinflammatory cytokine that induces adhesion molecules and chemokines, and plays an important role in UVB-induced inflammation and apoptosis in human skin [10]. It is also involved in UV-induced immunosuppression via its effect on Langerhans cell migration [11]. On the other hand, IL-10, a type 2 cytokine, is a mediator in the induction of systemic immunosuppression following UV exposure [12], [13]. IL-10 is found in the serum of UV-irradiated mice, and treatment of UV-irradiated mice with anti-IL-10 blocks the induction of immunosuppression [14]. IL-10 production and secretion in keratinocytes are triggered by UV-induced CPD formation [15]. Further, a cytokine cascade involving IL-10 results in prostaglandin E2 (PGE_2_) and IL-4 release upstream of UV-induced immunosuppression [16]. PGE_2_, which is an oxygenated metabolite of arachidonic acid produced by cyclooxygenase (COX) and secreted abundantly by keratinocytes upon UV exposure, is an important mediator of not only UV-induced immunosuppression, but also the sunburn response [17], [18]. Conversely, IL-12 is one of the major players involved in orchestrating both innate and acquired immune responses, and strongly induces the production of interferon (IFN)-γ from natural killer (NK) cells, leading to the development of T helper 1 responses [19], [20]. In addition, IL-12 suppresses UV-induced IL-10 and TNF-α production in keratinocytes, thereby ameliorating UV-induced immunosuppression [21], [22]. Furthermore, Schwarz et al. [23] reported that IL-12 could protect keratinocytes from apoptosis caused by DNA-damaging UV radiation by inducing DNA repair, and they also showed that nucleotide excision repair (NER) is modulated by IL-12.

Recently, Loser et al. [24] reported that epidermal receptor activator of nuclear factor κB (RANK) ligand (RANKL), which is a member of the TNF family, is associated with UV-induced regulatory T (Treg) cells and immunosuppression. They indicated that UV irradiation induces expression of RANKL in keratinocytes, and then RANKL activates its receptor RANK in epidermal Langerhans cells, leading to induction of Treg cell proliferation. In addition, expression of RANKL in keratinocytes stimulates Langerhans cells to produce IL-10 [25].

**Figure 1:**
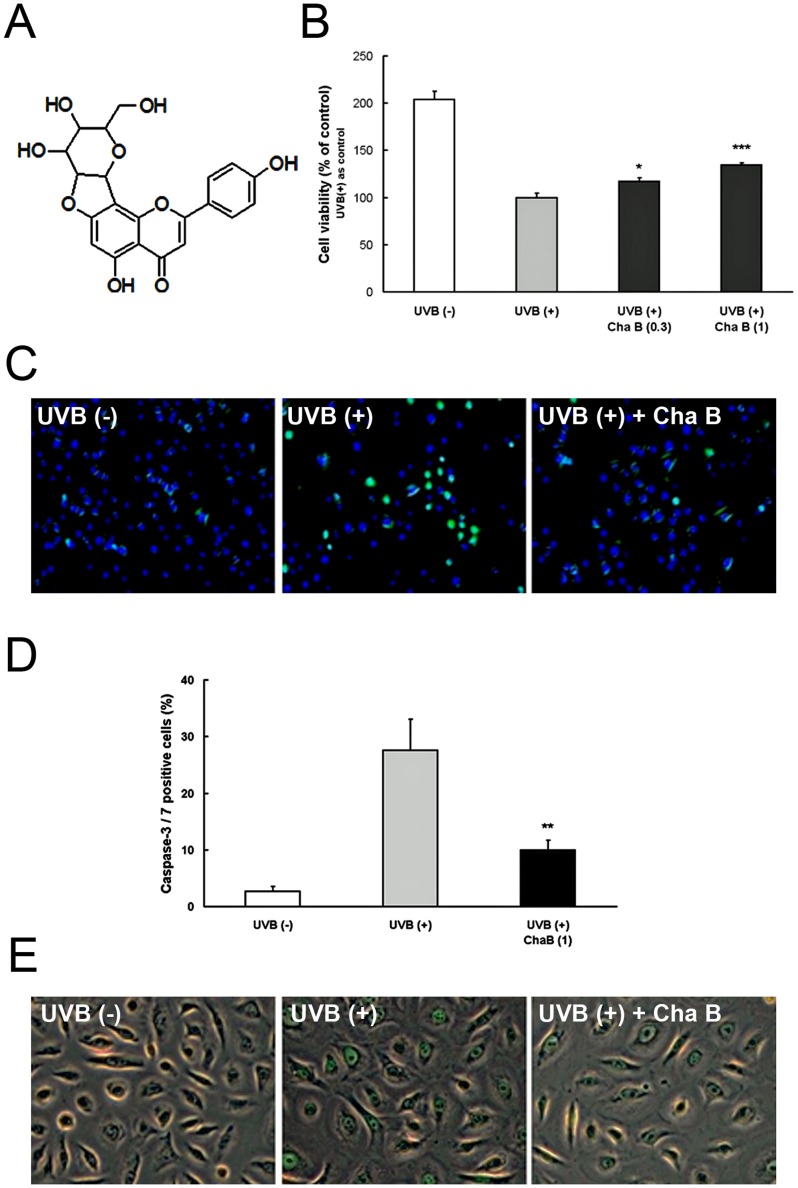
Chafuroside B attenuated DNA damage, cell damage, and apoptosis in UVB-exposed human keratinocytes. A Chemical structure of chafuroside B. B NHEK were irradiated with UVB (20 mJ/cm^2^), and then treated with chafuroside B (0.3 and 1 µM). Alamar blue assay was used to evaluate cell viability at 48 h after treatment. C, D NHEK were irradiated with UVB (20 mJ/cm^2^), and then treated with chafuroside B (1 µM). After 6 h, apoptotic cells were detected with CellEvent Caspase-3/7 Green Detection Reagent, which produces bright green fluorescence. Nuclei were stained using Hoechst 33342, exhibiting blue fluorescence. Numbers below the panel indicate percent of caspase-3/7-active cells detected in each population (100 cells, at least, were counted in each of the plates). E NHEK were irradiated with UVB (20 mJ/cm^2^), and then treated with chafuroside B (1 µM). After 24 h, CPD in genomic DNA was detected by an immunofluorescence method using FITC-labeled CPD specific antibodies. Cha B  =  Chafuroside B. All data are expressed as the mean ± sd (*n* = 3 or 4). *P<0.05, **P<0.01 and ***P<0.001 compared with UVB (+).

There has been considerable interest in the application of polyphenols for the prevention of UV-induced skin photodamage [26]. Polyphenols are a large family of naturally occurring plant products and are widely distributed in plant foods, such as fruits, vegetables, nuts, flowers, bark and seeds. Among them, green tea polyphenols, such as catechin and (–)-epigallocatechin-3-gallate (EGCG), have a variety of ameliorating effects on UV-induced skin damage [27]. Recently, a novel flavone *C*-glycoside polyphenol, chafuroside B (Fig. 1A), was isolated from oolong tea leaves, and such flavone *C*-glycoside found to have potent anti-inflammatory activity in experimental asthma/chronic obstructive pulmonary disease (COPD)-model rats [28], [29]. However, the effects of chafuroside B on skin function have not been examined.

In this study, we show that chafuroside B ameliorates UVB-induced cell damage by blocking UVB-induced apoptosis and DNA damage in normal human epidermal keratinocytes (NHEK). In addition, chafuroside B suppresses the production of UVB-induced immunosuppressive mediators, including IL-10, TNF-α and PGE_2_, and expression of RANKL in NHEK. These effects appear to be mediated at least in part through induction of IL-12 synthesis in NHEK.

## Materials and Methods

### Materials

Normal human epidermal keratinocytes (NHEK) were obtained from Kurabo Co. Ltd. (Japan). Serum-free keratinocyte growth medium (KGM) containing low calcium (0.06 mM), bovine pituitary extract (BPE) and epidermal growth factor (EGF), insulin, hydrocortisone and antibiotics (gentomycin/amphotericin) were purchased from GIBCO BRL (USA). Chafuroside B was isolated from oolong tea leaves and its structure was confirmed by spectroscopic analysis [28].

### Cell culture

NHEK were seeded at a density of 3–5×10^4^ cells/cm^2^ into 75 cm^2^ cell culture flasks, and cultured in KGM at 37°C under an atmosphere of 5% CO_2_ in air. Third or fourth passage cells were used for the experiments. Chafuroside B was dissolved in DMSO and diluted with the medium to appropriate concentrations; the final volume of DMSO was adjusted to 0.1% (v/v). In experiments to evaluate the effects of chafuroside B, NHEK were cultured in keratinocyte test medium (KTM: KGM without BPE and EGF).

### Cell viability assay

NHEK were grown to subconfluence using KGM in 35 mm dishes. The cultures were washed with KTM, grown in KTM for 1 day, irradiated through PBS with UVB (20 mJ/cm^2^), and then treated with chafuroside B for 48 h in KTM. After treatment, cell viability was evaluated by Alamar blue assay.

### Apoptosis assay

NHEK were grown to subconfluence using KGM. The cultures were washed with KTM, grown in KTM for 1 day, irradiated through PBS with UVB (20 mJ/cm^2^), and then treated with chafuroside B for 6 h in KTM. After treatment, cells were loaded with 5 µM CellEvent™ Caspase-3/7 Green Detection Reagent (Life Technologies, USA). The cells were fixed and imaged with a ZEISS Axio Observer Z1 fluorescence microscope.

### Immunofluorescence detection of CPD generation in DNA

NHEK were grown to subconfluence using KGM. The cultures were washed with KTM, grown in KTM for 1 day, irradiated through PBS with UVB (20 mJ/cm^2^), and then treated with chafuroside B for 24 h in KTM. After treatment, CPD in keratinocytes was evaluated with an OxiSelect™ Cellular UV-Induced DNA Damage Staining Kit (CPD) (Cell Biolabs Inc, USA), according to the manufacturer’s protocol. Stained cells were imaged with a fluorescence microscope.

### TNF-α, PGE_2_, and IL-10 production assay

NHEK were grown to subconfluence using KGM. The cultures were washed with KTM, grown in KTM for 1 day, irradiated through PBS with UVB (20 mJ/cm^2^), and then treated with chafuroside B for 48 h in KTM. After the treatment, the culture medium was collected and centrifuged. TNF-α, PGE_2_, and IL-10 in the supernatants were analyzed using a Human TNF-α Immunoassay Kit (R&D Systems Inc., USA), PGE_2_ EIA Kit (Enzo Life Sciences Inc., USA), and High Sensitivity IL-10 Human ELISA Kit (Abcam plc, USA), respectively.

### IL-12 production assay

NHEK were grown to subconfluence using KGM. The cultures were washed with KTM, grown in KTM for 1 day, and then treated with chafuroside B for 72 h in KTM. The culture medium was collected and centrifuged. IL-12 (p70) in the supernatants was analyzed using a Human IL-12 Quantikine HS ELISA Kit (R&D Systems Inc., USA).

### RNA extraction and analyses of mRNA expression levels of IL-12 and RANKL

After each treatment, cells were harvested and homogenized in Lysis buffer. Total RNAs were extracted from harvested keratinocytes using a RNeasy® Mini Kit (Qiagen, USA), according to the manufacturer’s protocol. The obtained total RNAs were used for cDNA production with a Transcriptor First Strand cDNA Synthesis Kit (Roche Applied Science, USA) according to the manufacturer’s protocol. The cDNAs were PCR-amplified with IL-12 and RANKL specific primers and β-actin primer as the internal control (Sigma-Aldrich Inc., USA). Fluorograms are shown after 45 cycles of PCR for IL-12, RANKL and β-actin. The sequences of the primer pairs, 5’ and 3’, were as fellows. IL-12: cactcccaaaacctgctgag and caatctcttcagaagtgcaagg; RANKL: tgattcatgtaggagaattaaacagg and gatgtgctgtgatccaacga; β-actin: ccaaccgcgagaagatga and ccagaggcgtacagggatag.

### Statistical analysis

All data were expressed as the mean ± SD. To evaluate the statistical significance of differences in multiple-group comparisons with a control group, we used Dunnett’s test or Steel’s test after Bartlett’s test. Differences were considered significant at *P*<0.05.

## Results

### Chafuroside B attenuated UVB-induced cell damage and apoptosis in human keratinocytes

To determine whether UVB-induced NHEK cell damage is attenuated by chafuroside B, we investigated the effect of chafuroside B on UVB-induced cell damage in cultured NHEK cells by means of Alamar blue assay. As shown in Fig. 1B, cell viability was decreased to about 50% by UVB radiation (20 mJ/cm^2^), but post-treatment with chafuroside B at 0.3 or 1 µM for 48 h significantly reduced the decrease of cell viability. Next, to assess the effect of chafuroside B on UVB-induced keratinocyte apoptosis, apoptosis was evaluated by measuring caspase-3/7 activity in NHEK. NHEK were exposed to UVB (20 mJ/cm^2^) and immediately post-treated with chafuroside B after UVB irradiation. UVB exposure resulted in the induction of keratinocyte apoptosis after 6 h (Fig. 1C; Middle), compared with non irradiated cells (Fig. 1C; Left). Chafuroside B at 1 µM decreased the number of caspase-3/7 active cells, suggesting that chafuroside B might attenuate apoptosis of UVB-exposed NHEK cells (Fig. 1C; Right). Actually, UVB radiation (20 mJ/cm^2^) increased number of caspase-3/7 active cells, compared with non UVB-treated cells. This increase of number was significantly suppressed by post-treatment with chafuroside B; 28% of UVB radiation cells were caspase-3/7 positive, whereas only 10% of chafuroside B (1 µM)-treated cells were positive (Fig. 1D).

### Chafuroside B induced repair of UVB-induced CPD generation in human keratinocytes

To determine whether chafuroside B induces repair of UVB-induced DNA damage in NHEK, we investigated the effect of chafuroside B on CPD formation and repair. For this purpose, UVB-irradiated (20 mJ/cm^2^) cells were immediately treated with or without chafuroside B for 24 h, and CPD-positive cells were evaluated with CPD-specific antibody. No CPD-positive cells were detected in non-UVB-irradiated cells (Fig. 1E; Left). And, we confirmed that CPD-staining intensity was weaker in NHEK post-treated with chafuroside B at 1 µM for 24 h (Fig. 1E; Right), as compared with untreated cells (Fig. 1E; Middle), suggesting that chafuroside B might accelerate the repair of UVB-induced CPD in NHEK.

### Chafuroside B suppressed the production of TNF-α induced by UVB radiation in human keratinocytes

**Figure 2:**
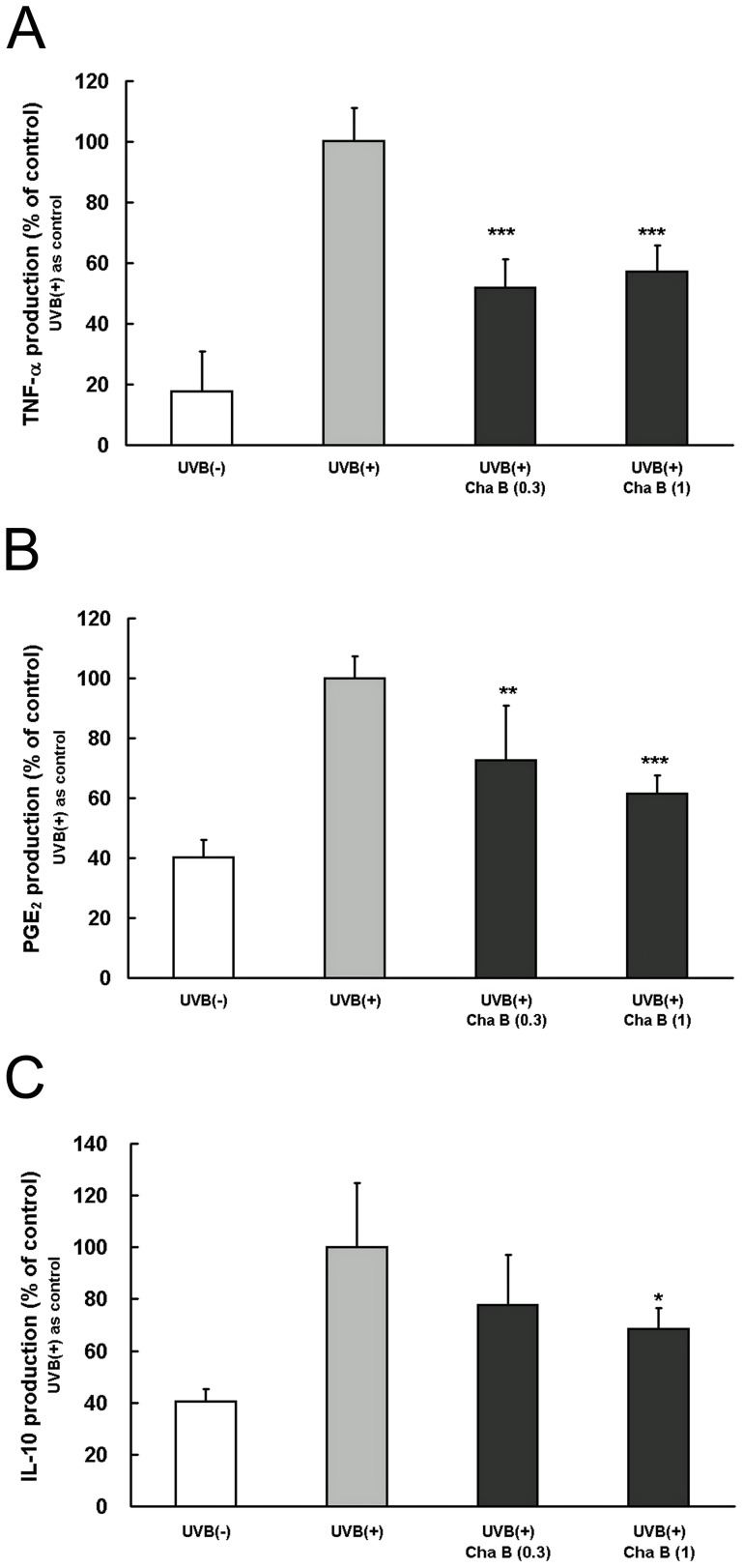
Chafuroside B decreased the UVB-induced production of TNF-α, PGE_2_, and IL-10 in human keratinocytes. NHEK were irradiated with UVB (20 mJ/cm^2^), and then treated with chafuroside B (0.3 and 1 µM). After 48 h, supernatants were collected and the levels of TNF-α, PGE_2_, and IL-10 were evaluated by ELISA. (A) TNF-α. (B) PGE_2_. (C) IL-10. Cha B  =  Chafuroside B. All data are expressed as the mean ± sd (*n* = 4). *P<0.05, **P<0.01 and ***P<0.001 compared with UVB (+).

TNF-α is a cytokine associated with UVB-induced immunosuppression, and release of TNF-α from keratinocytes is stimulated by UV radiation [10]. Therefore, we next investigated whether chafuroside B suppresses UVB-induced TNF-α production in cultured NHEK. UVB radiation (20 mJ/cm^2^) increased production of TNF-α by about 6-fold over 48 h, compared with non UVB-treated cells. This increase of TNF-α was significantly suppressed by post-treatment with chafuroside B at 0.3 and 1 µM (Fig. 2A).

### Chafuroside B suppressed the production of PGE_2_ induced by UVB radiation in human keratinocytes

PGE_2_ is associated with UVB-induced immunosuppression, and release of PGE_2_ from keratinocytes is stimulated by UV radiation [30]. Therefore, we examined the suppressive effect of chafuroside B on UVB-induced PGE_2_ production in cultured NHEK. UVB radiation (20 mJ/cm^2^) increased production of PGE_2_ by about 2.5-fold over 48 h, compared with non UVB-treated cells. This increase was significantly suppressed by post-treatment with chafuroside B at 0.3 and 1 µM (Fig. 2B).

### Chafuroside B suppressed the production of IL-10 induced by UVB radiation in human keratinocytes

IL-10 is a cytokine associated with UVB-induced immunosuppression, and its release is stimulated by UV-induced DNA damage [13]. Therefore, we investigated whether chafuroside B suppresses UVB-induced IL-10 production in cultured NHEK. UVB radiation (20 mJ/cm^2^) increased production of IL-10 by about 2.5-fold over 48 h, compared with non UVB-treated cells. This increase was significantly suppressed by post-treatment with chafuroside B at 1 µM (Fig. 2C).

### Chafuroside B enhanced IL-12 mRNA expression and production in human keratinocytes

**Figure 3:**
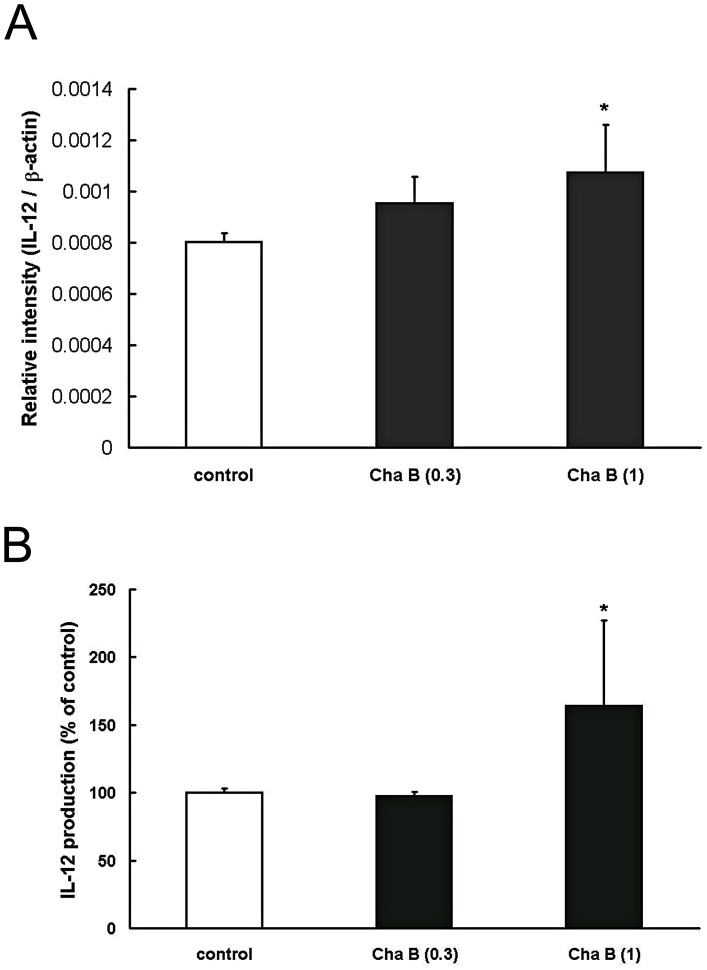
Chafuroside B enhanced IL-12 mRNA expression and production in human keratinocytes. A NHEK were treated with chafuroside B (0.3 and 1 µM). The cells were harvested after 3 h and IL-12 mRNA was quantitated by means of RT-PCR. B NHEK were treated with chafuroside B (0.3 and 1 µM). After 72 h, supernatants were collected and IL-12 was evaluated by means of ELISA. Cha B  =  Chafuroside B. All data are expressed as the mean ± sd (*n* = 3 or 4). *P<0.05 compared with the control.

IL-12 ameliorates UV-induced apoptosis and DNA damage of keratinocytes, and blocks UV-induced IL-10 and TNF-α production in keratinocytes [21], [23]. Further, human keratinocytes are known to have the capacity to release IL-12 [31]. Therefore, in order to determine whether the ameliorating effect of chafuroside B on UVB-induced DNA damage and IL-10 and TNF-α induction is mediated through IL-12 induction, we examined the effect of chafuroside B on IL-12 mRNA and production in human keratinocytes. As shown in Fig. 3A, RT-PCR showed that chafuroside B at 1 µM increased the IL-12 mRNA level during 3 h incubation, as compared with the control. Next, NHEK were treated with chafuroside B for 72 h, and the IL-12 concentration in the culture supernatant was measured by ELISA. As shown in Fig. 3B, chafuroside B at 1 µM increased the IL-12 production by about 1.6-fold during 72 h incubation, as compared with the control.

### Chafuroside B suppressed the mRNA expression of RANKL induced by UVB radiation in human keratinocytes

**Figure 4:**
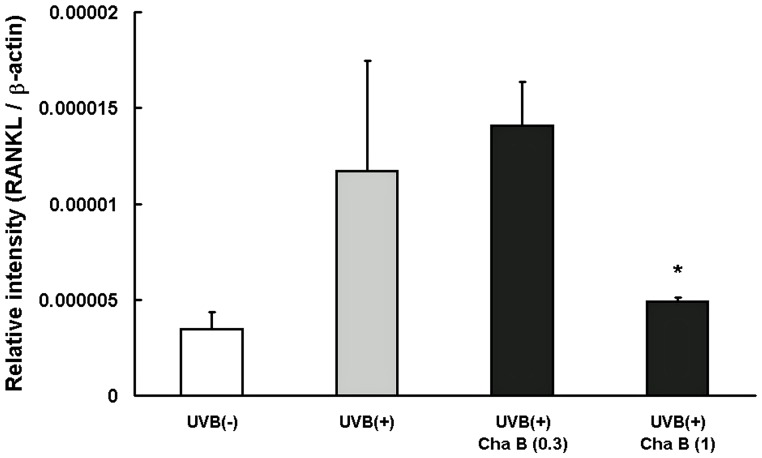
Chafuroside B decreased the mRNA expression of RANKL induced by UVB radiation in human keratinocytes. NHEK were irradiated with UVB (20 mJ/cm^2^), and then treated with chafuroside B (0.3 and 1 µM). The cells were harvested after 24 h and RANKL mRNA was evaluated by means of RT-PCR. Cha B  =  Chafuroside B. All data are expressed as the mean ± sd (*n* = 3 or 4). *P<0.05 compared with UVB (+).

Expression of RANKL, a UVB-induced immunosuppression mediator, in keratinocytes is increased in response to UV irradiation or inflammation [24]. Therefore, we investigated whether chafuroside B inhibits the UVB-induced expression of RANKL mRNA in cultured NHEK by means of RT-PCR. As expected, UVB radiation (20 mJ/cm^2^) up-regulated RANKL mRNA expression at 24 h. The up-regulation was significantly suppressed by post-treatment with chafuroside B at 1 µM (Fig. 4).

## Discussion

In this study, we found that chafuroside B attenuated UVB-induced apoptosis and enhanced DNA repair in UVB-exposed NHEK. Furthermore, chafuroside B suppressed production of IL-10, TNF-α and PGE_2_, and mRNA expression of RANKL, all of which are UVB-induced immunosuppression related mediators, in UVB-exposed NHEK. These ameliorating effects of chafuroside B on UVB-induced DNA damage, apoptosis and generation of immunosuppression related mediators appeared to be mediated at least in part through IL-12 induction in NHEK. About 90% of skin cancer cases have been attributed to solar UV radiation, particularly its UVB component, which is well absorbed by cellular DNA [32]. UVB radiation can penetrate the epidermis of the skin, inducing both direct and indirect DNA-damaging effects. As a direct effect, UVB induces generation of CPD, which causes DNA mutation leading to tumor initiation, transcriptional modulation of genes involved in tumor promotion, and activation of several signal transduction pathways [33]. In addition, UVB indirectly damages DNA through generation of reactive oxygen species (ROS), which facilitate the oxidation of DNA [34]. UVB-induced apoptosis has been recognized as a protective mechanism, because it contributes to removal of cells carrying DNA damage, thereby preventing malignant transformation [35]. Our data showed that chafuroside B suppressed UVB-induced CPD generation in DNA of keratinocytes by promoting repair of damaged DNA, thereby leading to the attenuation of UVB-induced apoptosis and cell death.

NER is a major endogenous mechanism for repair of UV-induced DNA lesions in mammalian cells. The cytokine IL-12 reduces the amount of DNA damage in skin [23], and this effect was not observed in *Xpa* knockout mice, which lack functional NER owing to a mutation in the *Xpa* gene [36]. Therefore, IL-12 may reduce CPD via induction of NER. Further, UVB-induced inflammatory responses, such as production of cytokines, are causally related to UVB-induced DNA damage. The levels of inflammatory biomarkers, such as PGE_2_, COX-2, TNF-α, IL-1β, and IL-6, were higher in UVB-exposed skin of IL-12 knockout mice than in that of wild-type mice when levels of UV-induced DNA damage were elevated, suggesting that IL-12 knockout mice are more susceptible to UV-induced inflammation because of defective repair of UVB-induced DNA damage [37]. Likewise, the levels of inflammatory biomarkers, such as PGE_2_, TNF-α, and IL-1β, were also elevated when levels of UV-induced DNA damage were higher, and subsequently declined when the levels of DNA damage decreased [37]. In this study, we found that chafuroside B enhanced IL-12 mRNA expression and production in cultured keratinocytes, suggesting that the decrease of UVB-induced CPD generation by chafuroside B is mediated at least in part by IL-12 via the NER mechanism. IL-12 has been reported to antagonize UV-induced immunosuppression [38]. In addition, IL-12 is able to break established immunotolerance, which is mediated through Treg cells [39], [40]. Conversely, impairment of the immune system by UVB is mediated in large part by the immunosuppressive cytokine IL-10, the release of which is induced by UVB [14]. Our results show that chafuroside B suppressed UVB-induced IL-10 production in NHEK cells. IL-10 production in keratinocytes is triggered by UV-induced CPD generation, and inhibited by IL-12 [19], [21]. Accumulated evidence indicates that UV-induced immunosuppression is mediated at least in part by an increase in the production of IL-10 and a reduction in IL-12 [21]. Here, we found that chafuroside B changes the balance between these two cytokines, reducing IL-10 production and enhancing IL-12 production.

TNF-α is also involved in UVB-induced immunosuppression by blocking Langerhans cell migration and reducing the cell density in humans [41]. The migration of Langerhans cells is an initial event in the sensitization phase of immunosuppression. We showed that chafuroside B suppressed not only IL-10 production, but also TNF-α production in UVB-exposed keratinocytes. UVB-induced TNF-α production is blocked by IL-12 in keratinocytes and fibroblasts [22], suggesting that the suppressing effect of chafuroside B on TNF-α production is also involved in enhancement of IL-12 production. Keratinocyte-derived TNF-α mediates UVB-induced PGE_2_ release via TNF-α receptor on the keratinocyte cell surface in an autocrine manner [42]. Many studies have shown that nonsteroidal anti-inflammatory drugs (NSAIDs), including indomethacin, can reverse the immunosuppressive effect of UV radiation by suppressing production of prostanoids such as PGE_2_ through COX inhibition [43]. Further, PGE_2_ is released from keratinocytes exposed to UV radiation [30]. We found that chafuroside B inhibits the release of PGE_2_ by UVB-exposed keratinocytes.

Chafuroside B also suppressed the increase of RANKL mRNA expression in UVB-irradiated cultured human keratinocytes. It has been reported that RANKL signaling regulates the proliferation of Treg cells in peripheral organs via activation of RANK expressed in epidermal Langerhans cells [24]. Administration of anti-RANKL antibody has been shown to abolish UV-induced immunosuppression in mice [43]. RANK is also expressed in keratinocytes of mammalian skin, and RANK transgenic mouse epidermis was greatly thickened, suggesting that RANK activates cell proliferation in epidermis [44]. RANKL expression is also markedly increased in the epidermis of patients with psoriasis, which is a chronic inflammatory skin disease characterized by hyperproliferation of keratinocytes [45]. Thus, RANKL and its receptor RANK are involved in regulation of immune and proliferative responses in skin epidermis. On the other hand, it has been reported that RANK may be involved in the development and maintenance of melanoma-initiating cells and possibly also in metastatic spreading [46]. Hence, factors that modulate the RANKL-RANK system, such as chafuroside B, can be considered as candidates for pharmacological application to treat skin disorders.

In conclusion, we found that chafuroside B ameliorates UVB-induced apoptosis and DNA damage in keratinocytes. Further, chafuroside B suppresses the generation of IL-10, TNF-α, PGE_2_, and RANKL, all of which are UVB-induced immunosuppression related mediators, in keratinocytes. These effects of chafuroside B appear to be mediated at least in part through IL-12 induction in keratinocytes. Because oolong tea, which contains chafuroside B, could easily be consumed as a dietary supplement for skin photoprotection, these findings suggest that not only topical treatment but also routine consumption of chafuroside B in tea may provide a degree of protection against the harmful effects of solar UV radiation in humans. A study of the in vivo effects of chafuroside B seems warranted.
